# Three-Dimensional Reconstruction and Scour Volume Detection of Offshore Wind Turbine Foundations Based on Side-Scan Sonar

**DOI:** 10.3390/s26020386

**Published:** 2026-01-07

**Authors:** Yilong Wang, Lijia Tao, Mingxin Yuan, Jingjing Yang

**Affiliations:** 1 School of Mechanical Engineering, Jiangsu University of Science and Technology, Zhenjiang 212100, China; 231210201420@stu.just.edu.cn (Y.W.); mxyuan78@just.edu.cn (M.Y.); 241210201202@stu.just.edu.cn (J.Y.); 2 Industrial Technology Research Institute (Advanced Metal Materials), Jiangsu University of Science and Technology, Zhangjiagang 215600, China

**Keywords:** offshore wind turbine foundations, side-scan sonar, three-dimensional reconstruction, volume detection, AlphaShape algorithm

## Abstract

To enable timely, effective, and high-accuracy detection of scour around offshore wind turbine pile foundations, this study proposes a three-dimensional reconstruction and scour volume detection method based on side-scan sonar imagery. First, the sonar images of pile foundations are preprocessed through grayscale conversion, binarization, and region expansion and merging to obtain an effective grayscale representation of scour pits. An optimized Shape-from-Shading (SFS) method is then applied to reconstruct the three-dimensional geometry from the effective grayscale map, generating point cloud data of the scour pits. Subsequently, the point cloud data are filtered using curvature and normal vector constraints, followed by depth-based *z*-axis descent detection, clustering, and morphological restoration to extract individual scour pit point clouds. Finally, a weight-corrected AlphaShape algorithm is employed to accurately calculate the volume of each scour pit. Numerical experiments involving five simulated scour scenarios across three types demonstrate that the proposed method achieves accurate identification and extraction of scour pit point clouds, with an average volume measurement accuracy of 97.495% compared with theoretical values. Field measurements in real-world environments further validate the effectiveness of the proposed method for practical scour volume detection around offshore wind turbine foundations.

## 1. Introduction

With the rapid expansion of global offshore wind power installations [1,2,3], the structural health monitoring of pile foundations has become a critical factor constraining the sustainable development of offshore wind energy. Studies have shown that scour pits formed around pile foundations as a result of seabed erosion can significantly compromise structural stability. Traditional inspection methods, such as manual underwater measurements, are highly dependent on water visibility and suffer from limited quantitative accuracy [4]. Although multibeam sonar can achieve millimeter-level detection accuracy, its operation is complex and requires multi-angle data acquisition around the target, making it unsuitable for large-scale and routine monitoring of offshore wind farms [5,6]. Despite the rapid growth of the offshore wind industry over the past decade, many of the earliest large-scale wind farms are now approaching the end of their initial service life, shifting industry priorities toward full life-cycle operation and maintenance [7,8]. Therefore, in the maintenance of offshore wind turbine foundations, an urgent technical challenge lies in efficiently and accurately acquiring underwater scour conditions and related scour parameters around pile foundations.

Compared with the high cost of physical experiments, numerical simulation has attracted increasing attention for scour assessment around pile foundations, as it can incorporate multiple factors, such as wave parameters and sediment characteristics, enabling rapid and cost-effective evaluation. Mingming Liu et al. [9] numerically investigated steady-flow-induced scour around rectangular and square submarine caissons and revealed that the scour mechanism is strongly influenced by the incident flow angle: perpendicular flows induce horseshoe-vortex scour, whereas at an incident angle of 45°, local flow contraction intensifies side-edge velocities, becoming the dominant scour mechanism. Although this study provides valuable theoretical insights for scour protection design, it focused primarily on the influencing mechanisms and did not include quantitative calculations of scour volume under different conditions. Chong Sun et al. [10] proposed an empirical model for scour induced by Darrieus-type tidal turbine systems to predict the maximum scour depth and scour profiles along the turbine centerline. However, the analysis was limited to scour depth estimation and did not offer detailed quantitative measures of overall scour severity. Chenghao Zhu et al. [11] developed a coupled CFD–FEM–scour model (CFD-FEM-SM) to integrate the scour process with seabed mechanical responses, investigating the effects of wave- and current-induced seabed behavior on local scour. Nevertheless, these effects were characterized only in terms of scour depth, without more comprehensive numerical descriptions of scour extent or volume. Overall, current scour simulation approaches rely heavily on empirical sediment transport and turbulence models, meaning that their accuracy is constrained by model assumptions and parameter uncertainty, and remains insufficient for the precise scour assessment required in pile foundation maintenance. Therefore, quantitative detection based on physical sensing techniques is currently regarded as the most reliable solution.

With advancements in sonar technology, sonar-based scour detection for pile foundations has attracted increasing attention in recent years. Xiao Zhiguang et al. [12] employed side-scan sonar to investigate the in-service conditions of subsea pipelines, as well as the surrounding seabed topography and geomorphology. By integrating hydrodynamic conditions with sediment engineering properties, they analyzed the sonar image characteristics of unscoured seabeds, locally scoured seabeds, collapsed depressions, and partially exposed suspended pipelines, thereby confirming the feasibility of side-scan sonar for pipeline safety assessment. However, the analysis relied primarily on manual interpretation to assess scour severity, lacking quantitative scour metrics. Zhang Wenpeng [13] utilized both multibeam and side-scan sonar technologies to conduct a comprehensive survey of turbine locations, substation foundations, and cable routes in offshore wind farms, identifying seabed scour deformation and cable exposure. Nonetheless, the study did not calculate the actual volume of individual scour pits, which limits accurate evaluation of localized scour risk. Yulin Tang et al. [14] applied an improved U-Net architecture for semantic segmentation of sonar images, enabling two-dimensional detection of underwater scour and bridge structural damage. However, this approach provides only planar location and depth-related information, without three-dimensional volume estimation, and its applicability to scenarios involving multiple scour pits has not been validated. Ruth Durán et al. [15] combined multibeam sonar, side-scan sonar, and sediment data to investigate extensive seabed erosion caused by bottom trawling in the northwestern Mediterranean. Their assessment was largely qualitative, relying on discrete sampling and morphological descriptions, and lacked precise quantification of scour volume variations. Wang Hengbo [16] employed an integrated suite of single-beam sonar, multibeam sonar, side-scan sonar, and sub-bottom profiling to examine a submarine water supply pipeline in Hangzhou Bay, identifying seabed scour and micro-topographic features such as pipeline trenches. Although suspended pipeline height was reported, more detailed quantitative characterization of scour distribution along the pipeline was not provided.

In summary, although existing scour detection approaches based on multibeam sonar, side-scan sonar, and related techniques can clearly delineate scour regions around pile foundations, they remain incapable of accurately quantifying the severity of scour at offshore wind turbine foundations. This limitation hinders the provision of reliable technical support for refined operation and maintenance strategies. To address this issue, the present study utilizes high-resolution side-scan sonar imagery of seabed pile foundations and proposes a complete workflow for precise scour assessment. First, pile foundation regions are identified and extracted from the sonar images. Subsequently, the Shape-from-Shading (SFS) method is applied to reconstruct the three-dimensional structure of the extracted regions, generating the corresponding 3D point cloud of the pile foundations. The point cloud is then processed through filtering, depth-based descent detection, clustering, and morphological restoration to obtain the final scour pit point clouds. Finally, a weight-enhanced AlphaShape algorithm is employed to accurately calculate the volume of each individual scour pit.

## 2. Scour Pit Identification and Extraction from Side-Scan Sonar Images of Pile Foundations

### 2.1. Binarization of Side-Scan Sonar Images of Pile Foundations

**Figure 1:**
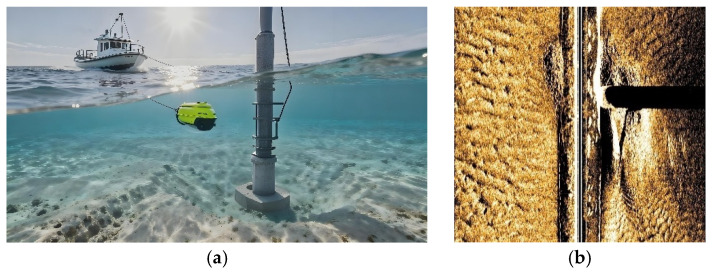
Pile foundation detection based on side-scan sonar: (**a**) pile foundation inspection method; (**b**) side-scan sonar image of pile foundation.

Scour detection of pile foundations is commonly conducted using a towfish-mounted side-scan sonar deployed from a survey vessel, as illustrated in Figure 1a. The corresponding side-scan sonar image of the pile foundation is shown in Figure 1b.

**Figure 2:**
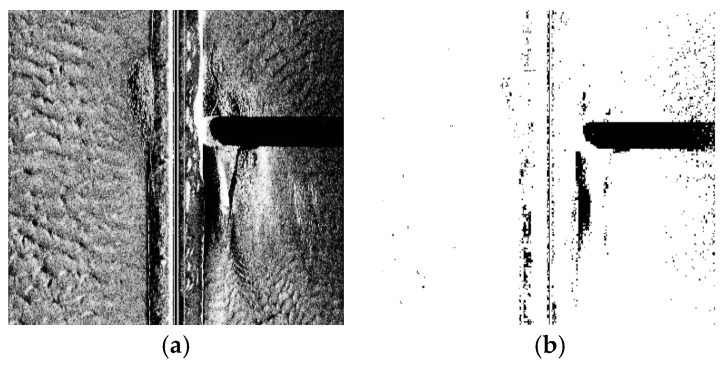
Binarization of the side-scan sonar image: (**a**) grayscale image; (**b**) binary image.

To enable accurate identification of pile foundation regions in subsequent processing of side-scan sonar images, the RGB sonar image is first converted into a grayscale image using the grayscale operation defined in Equation (1), resulting in the grayscale image shown in Figure 2a [17].

(1) $$I=0.2989I_{R}+0.5870I_{G}+0.1140I_{B}$$

where *I_R_*, *I_G_*, and *I_B_* denote the red, green, and blue pixel values of a given pixel, respectively, and I represents the resulting grayscale value of that pixel.

Since the boundary between the pile foundation region and the background is often ambiguous in the grayscale image, the image is further converted into a binary image, as shown in Figure 2b [18]. This binarization enhances the separation between the pile foundation region and the background, thereby facilitating the subsequent extraction of scour pit areas.

### 2.2. Scour Pit Extraction Based on Connected Components and Regional Features

To effectively detect scour pit regions, multiple connected components are first identified in the binary image containing the scour pits [19]. If there exists a path in the image consisting of *n* pixels with a value of 255, denoted as (*p*_1_, *p*_2_, …, *p_n_*), and the distance between adjacent pixels is less than a specified threshold, then the pixels *p*_1_ and *p_n_* are defined as connected:

(2) $$\forall i\in \left[2,n\right],\;\left\|p_{i}-p_{i-1}\right\|\leq \epsilon$$

where ‖·‖ denotes the Euclidean distance, and *ϵ* represents the neighborhood distance threshold.

After identifying multiple connected components, the pile foundation region is extracted by sequentially filtering the components based on their size, centroid, and boundary characteristics. Since the size of each connected component corresponds to the number of pixels with a grayscale value of 255, filtering can be performed according to the sum of grayscale values of all pixels within each component. Components whose total grayscale value exceeds a specified threshold $S_{\mathrm{Min}}^{G}$ are retained to avoid inclusion of small noise points. Let $S_{i}^{G}$ denote the sum of grayscale values of all pixels in the *i*-th connected component. If $S_{i}^{G}$ satisfies the condition in Equation (3), the component is retained, thereby preventing extraction of insignificant noise.

(3) $$S_{i}^{G}\geq S_{\mathrm{Min}}^{G}$$

Since the central region of the side-scan sonar image corresponds to the seabed line, and the pile foundation region is located away from this area, centroid-based filtering is applied to the components retained after size-based filtering in order to preserve regions distant from the seabed line. Let *W* denote the width of the side-scan sonar image; then, the horizontal coordinate of the central column of the image is given by *c*:

(4) $$c=\frac{W}{2}$$

Let *c_x_* denote the horizontal coordinate of the centroid of a connected component retained after size-based filtering. If *c_x_* is sufficiently distant from the central coordinate *c*, as specified in Equation (5), the component is retained for further processing.

(5) $$||c_{x}-c||>\delta$$

where *δ* represents the centroid distance threshold. Its value is determined based on the spatial dimensions of a single scour pit in the simulated data. Specifically, the equivalent diameter of a single scour pit in the binary image is calculated, and *δ* is set slightly larger than this equivalent diameter to prevent the same scour pit from being erroneously segmented into multiple regions, while also avoiding the incorrect merging of adjacent regions.

**Figure 3:**
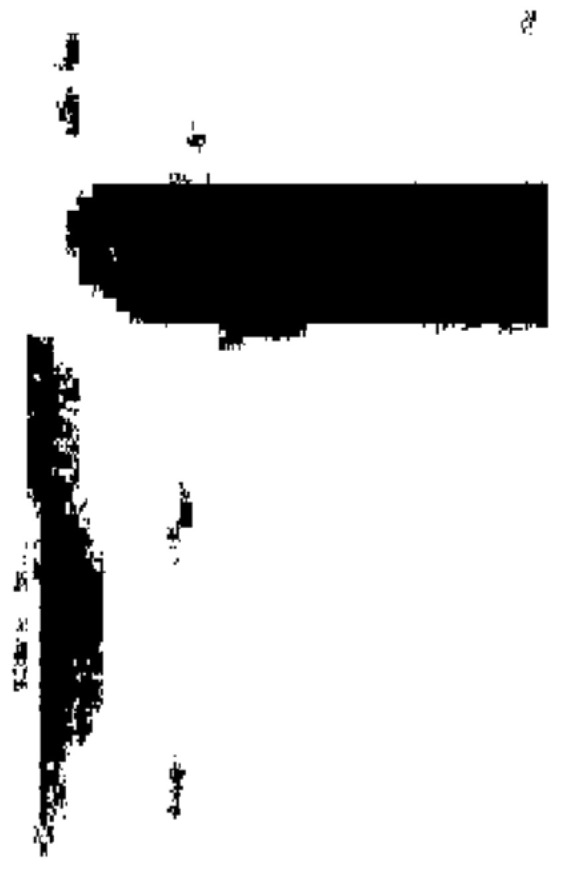
Final preserved connected domains.

To account for the possibility that some retained regions may still contain slender or irregular non-target areas, bounding-box filtering is performed on all connected components preserved after centroid filtering. Specifically, the maximum and minimum coordinates of each region, (*x*_max_, *y*_max_) and (*x*_min_, *y*_min_), are computed, and only the connected components that satisfy the following conditions are finally retained, as illustrated in Figure 3.

(6) $$\left\{\begin{array}{c} w=(x_{\max}-x_{\min})\geq w_{\min}\\ h=(y_{\max}-y_{\min})\geq h_{\min} \end{array}\right.$$

where *w* denotes the width of the bounding box, *h* represents the height of the bounding box, *w*_min_ is the minimum width threshold, and *h*_min_ is the minimum height threshold.

Let a total of m connected components be preserved after bounding-box filtering. These components are collectively denoted as ***R***(*m*).

### 2.3. Region Expansion and Merging of the Finally Preserved Connected Domains

Due to the presence of noise, uneven illumination, and the limitations of threshold-based segmentation [20], the preserved connected component set ***R***(*m*) may include only partial regions of the scour pits. To ensure complete extraction of the scour pit regions, a neighborhood search method is employed for region expansion. Specifically, for each connected component in ***R***(*m*), pixels with a value of 0 are searched within the neighborhood of every pixel in the component, provided that the distance between them does not exceed a specified threshold. The expansion is then performed iteratively for *k* iterations as follows:

(7) $$R^{k}(m)=R^{k-1}(m)\cup \{(x,y)|\exists (x\prime ,y\prime )\in R^{k-1}(m),∥(x,y)-(x\prime ,y\prime )∥\leq \omega \}$$

where (*x*′, *y*′) and (*x*, *y*) denote the coordinates of a pixel before and after expansion, respectively; ***R****^k^*(*m*) and ***R****^k^*^−1^(*m*) represent the sets of *m* connected domains after *k* and *k* − 1 iterations of expansion, respectively; and *ω* is the expansion threshold.

The iterative expansion is terminated once the following condition is satisfied, yielding the complete scour pit region.

(8) $$\min(\left\|\left(x,y)-(x\prime ,y\prime \right)\right\|)\geq d_{\max}$$

where *d*_max_ denotes the maximum allowable expansion distance.

All connected components in the set ***R***(*m*) are iteratively expanded to obtain the complete scour pit regions, forming a new set of connected components denoted as ***R***′(*m*).

After the expansion of the connected components, regional merging is performed to ensure that adjacent or overlapping components are effectively integrated. The method first computes the minimum boundary distance *d*(***R***′(*i*), ***R***′(*j*)) between any two connected components ***R***′(*i*) and ***R***′(*j*) in the set ***R***′(*m*).

(9) $$d(R^{\prime }(i),R^{\prime }(j))=\min(\left\|\left(x_{i},y_{i})-(x_{j},y_{j}\right)\right\|),(x_{i},y_{i})\in \partial R^{\prime }(i),(x_{j},y_{j})\in \partial R^{\prime }(j)$$

where ∂***R***′(*i*) and ∂***R***′(*j*) denote the sets of boundary points of the connected domains ***R***′(*i*) and ***R***′(*j*), respectively.

Next, it is determined whether ***R***′(*i*) and ***R***′(*j*) satisfy the merging condition, i.e., whether *d*(***R***′(*i*), ***R***′(*j*)) is less than or equal to the maximum allowable merging distance threshold *l*. If this condition is met, a new connected component ***R***′(*ij*) is obtained by merging ***R***′(*i*) and ***R***′(*j*) according to Equation (9); otherwise, ***R***′(*i*) and ***R***′(*j*) remain as two separate connected components.

(10) $$R^{\prime }\left(ij\right)=R^{\prime }\left(i\right)\cup R^{\prime }\left(j\right)$$

**Figure 4:**
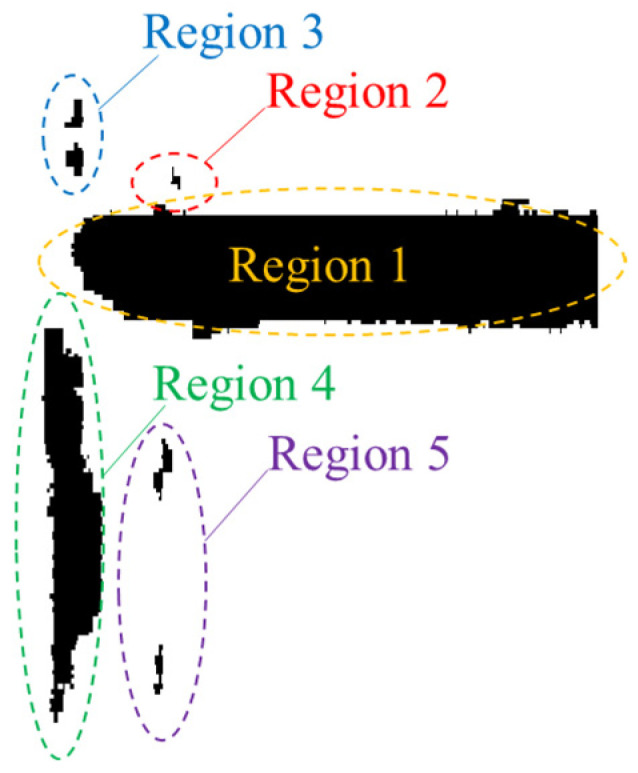
Final merged regions.

Through repeated region-merging operations, all individual connected components are consolidated, resulting in separate scour pit regions. Figure 4 shows the five independent scour pit regions obtained after performing region expansion and merging on the connected components in Figure 3.

**Figure 5:**
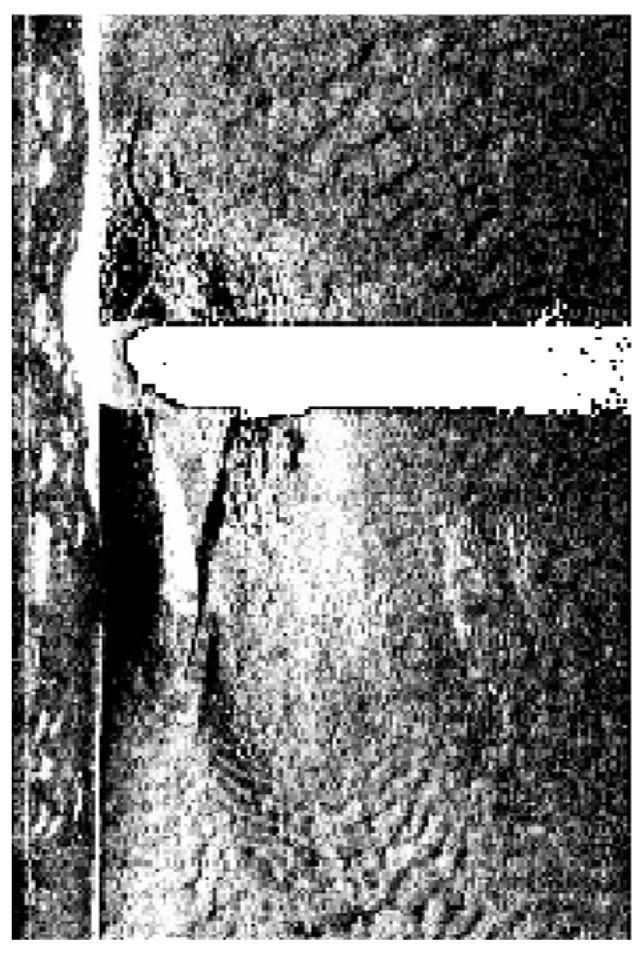
Effective grayscale image of pile-foundation scour pits.

Since the scour pit regions obtained after region expansion and merging may still contain non-target areas caused by pile-induced acoustic shadows, it is necessary to identify and remove these shadow regions to obtain valid scour pit areas. The procedure is based on the characteristics of side-scan sonar imaging, in which acoustic shadows typically appear as continuous connected regions with areas significantly larger than those of actual scour pits. In this study, candidate regions are ranked using connected component analysis, and the component with the largest area is identified as the acoustic shadow and subsequently removed. The selection of parameters in this method is not based on fixed values; instead, it is adaptively determined according to the relative area ranking, which improves the effectiveness of acoustic shadow removal. Furthermore, the 3D reconstruction of the pile-foundation side-scan sonar image is performed based on grayscale information. Therefore, the binary image with pile shadows removed is combined with the grayscale image of the scour pit regions, resulting in the effective grayscale image of the pile-foundation scour pits shown in Figure 5.

## 3. Three-Dimensional Reconstruction of the Effective Grayscale Image of Pile-Foundation Scour Pits

Since accurate calculation of scour pit volumes relies on three-dimensional point cloud data, it is necessary to perform 3D reconstruction of the obtained effective grayscale image of the pile-foundation scour pits to acquire the corresponding pile-foundation point cloud data. The Shape-from-Shading (SFS) method can infer the three-dimensional surface features of an object based on brightness variations within a two-dimensional image, in combination with an optical reflection model. Therefore, this study applies the SFS method to reconstruct the 3D geometry of the final effective grayscale image of the pile-foundation scour pits. In conventional SFS methods, the initial *z*-coordinate values of the pile image are randomly assigned, which can reduce the convergence speed during iterative reconstruction. To address this issue, the initial depth is generated using Gaussian kernel convolution, and brightness-driven adaptive parameters are constructed to optimize the SFS method.

### 3.1. Acquisition of Initial Depth Values for the z-Coordinate

Since the initial *z*-coordinate values in the pile-foundation image directly affect both the convergence efficiency of the depth iteration and the accuracy of the 3D reconstruction, this study optimizes the acquisition of the initial depth values as follows:

Let the grayscale value of a pixel (*x*″, *y*″) in the final effective grayscale image of the pile-foundation scour pits be *I* (*x*″, *y*″). The pixel value is first normalized as follows:

(11) $$I^{\prime }(x\prime \prime ,y\prime \prime )=\frac{I(x\prime \prime ,y\prime \prime )-I_{\min}}{I_{\max}-I_{\min}}$$

where *I*(*x*″, *y*″) and *I*′(*x*″, *y*″) represent the grayscale values of the pixel (*x*″, *y*″) before and after normalization, respectively, while *I*_min_ and *I*_max_ denote the minimum and maximum grayscale values of the final effective grayscale image of the pile-foundation scour pits.

Then, a Gaussian kernel *G_σ_*(*ξ*, *η*) is constructed for the image *I*′(*x*″, *y*″) as follows:

(12) $$G_{\sigma }(\xi ,\eta )=\frac{1}{2\pi \sigma^{2}}e^{-\frac{\xi^{2}+\eta^{2}}{2\sigma^{2}}}$$

where *σ* is the standard deviation, which determines the degree of smoothing; *ξ* and *η* represent the horizontal and vertical offsets from the center of the Gaussian kernel, respectively.

Finally, the initial depth *z*_0_ of the pixel (*x*″, *y*″) is obtained by performing a two-dimensional convolution of *I*′(*x*″, *y*″) with *G_σ_* (*ξ*, *η*):

(13) $$z_{0}=\int_{-\infty }^{+\infty }\int_{-\infty }^{+\infty }I^{\prime }(x\prime \prime -\xi ,y\prime \prime -\eta )G_{\sigma }(\xi ,\eta )d\xi d\eta$$

where (*x*″ − *ξ*, *y*″ − *η*) represents the pixel obtained by shifting the current pixel (*x*″, *y*″) by the Gaussian kernel center (*ξ*, *η*).

By repeating the above steps, the initial depth $z_{0}$ is calculated for each pixel. This initial depth provides a reasonable starting point for subsequent iterations, reducing the number of iterations required. Furthermore, it is closer to the true topography, thereby enhancing the accuracy of both the 3D reconstruction and the volume calculation of the effective grayscale image of the pile-foundation scour pits.

### 3.2. Three-Dimensional Reconstruction of the Effective Grayscale Image of Pile-Foundation Scour Pits Based on the SFS Method

The brightness of measured pile-foundation side-scan sonar images is typically influenced by both illumination conditions and the shape of the seabed surface. Therefore, it is necessary to establish the relationship between image brightness and seabed topography in order to accurately infer seabed depths from image brightness, thereby enabling precise reconstruction of the terrain surface.

To establish this relationship, assuming a linear correlation between image brightness and depth, the error function for a pixel (*x*″, *y*″) in the image can be expressed as:

(14) $$E(x\prime \prime ,y\prime \prime )=I(x\prime \prime ,y\prime \prime )-\left(s_{1}\frac{\partial z}{\partial x\prime \prime }+s_{2}\frac{\partial z}{\partial y\prime \prime }\right)$$

where *s*_1_ and *s*_2_ are the illumination parameters in the *x*-directions and *y*-directions, respectively; ∂*z*/∂*x*″ represents the slope of the terrain in the *x*-direction, and ∂*z*/∂*y*″ represents the slope of the terrain in the *y*-direction.

To obtain the final accurate depth for each pixel, this study performs iterative optimization of the initial depth based on the pixel-wise error function. A combination of gradient descent and constraints is employed to achieve the three-dimensional reconstruction of the effective grayscale image of the pile-foundation scour pits:

(15) $$z^{k+1}(x\prime \prime ,y\prime \prime )=z^{k}(x\prime \prime ,y\prime \prime )-\lambda E^{k}(x\prime \prime ,y\prime \prime )+\mu [z_{0}-z^{k}(x\prime \prime ,y\prime \prime )]$$

where *z^k^*(*x*″, *y*″) and *z^k^*^+1^(*x*″, *y*″) represent the depths of pixel (*x*″, *y*″) after the *k*-th and (*k* + 1)-th iterations, respectively; when *k* = 0, *z*^0^(*x*″, *y*″) = *z*_0_. *λ* is the gradient descent step size, determining the magnitude of depth adjustment during iteration; *μ* is the constraint weight, which regulates the balance between the current depth and the initial depth; *E^k^*(*x*″, *y*″) is the error function of pixel (*x*″, *y*″) at the k-th iteration, calculated as follows:

(16) $$E^{k}(x\prime \prime ,y\prime \prime )=I(x\prime \prime ,y\prime \prime )-\left(s_{1}\frac{\partial z^{k}}{\partial x\prime \prime }+s_{2}\frac{\partial z^{k}}{\partial y\prime \prime }\right)$$

The parameters λ and μ are determined as follows:

(17) $$\lambda =\frac{0.1}{\Delta I}$$

(18) μ=0.01ΔI

where Δ*I* denote the range of image brightness, that is, the difference between the maximum and minimum pixel grayscale values.

The convergence criterion of the SFS algorithm is that the absolute value of the error function *E^k^*(*x*″, *y*″) is less than a preset tolerance threshold *t*, or the iteration number *k* reaches the preset maximum *k*_max_, i.e.,:

(19) $$\left|E^{k}(x\prime \prime ,y\prime \prime )\right|<t\;\mathrm{OR}\;k\geq k_{\max}$$

**Figure 6:**
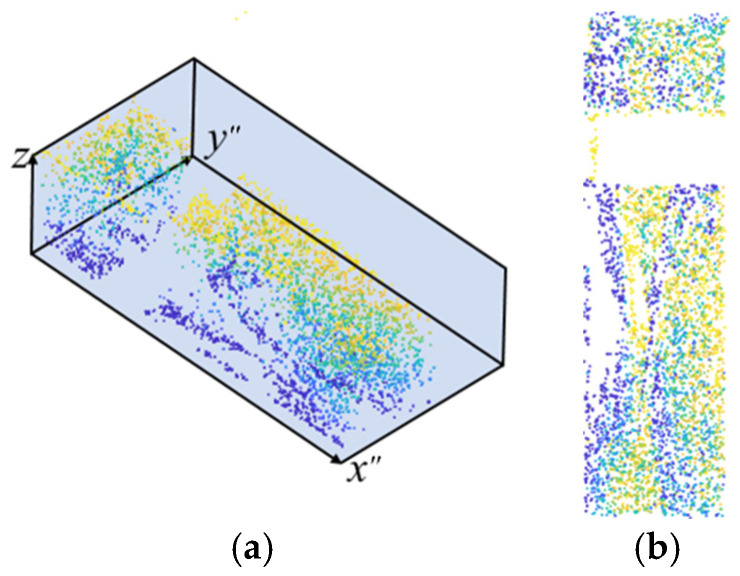
Point cloud of pile-foundation scour pits: (**a**) 3D view; (**b**) top view.

For intuitive visualization and extraction of the scour pits, colors are used to represent the depth of the point cloud, with darker colors indicating greater depth, as shown in Figure 6. Let a point in the point cloud after the *k*-th iteration be represented as *p =* (*x*″, *y*″, *z^k^*(*x*″, *y*″)); the set of pile-foundation scour-pit points in the figure is denoted as ***P***.

## 4. Scour-Pit Point Cloud Filtering and Morphological Restoration

### 4.1. Point Cloud Filtering and Extraction

**Figure 7:**
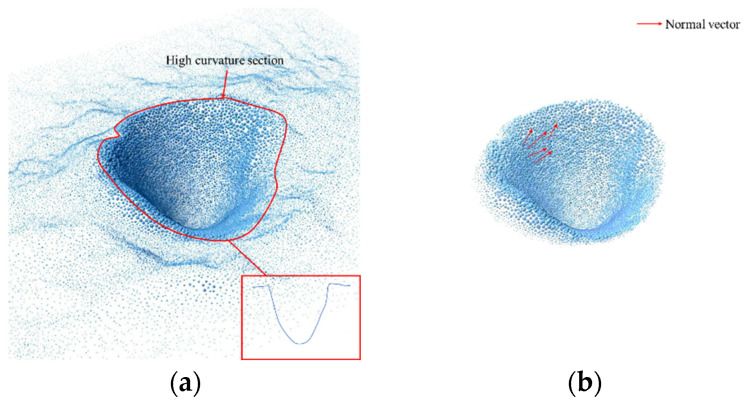
Scour-pit point cloud extraction: (**a**) point cloud filtering based on curvature; (**b**) point cloud filtering based on normal vectors.

To efficiently extract the scour pit point cloud from the pile-foundation point cloud, non-depressed regions must first be filtered out. Figure 7 illustrates the key steps of this process. Depressed and non-depressed regions in the pile-foundation point cloud exhibit significant differences in curvature and normal vectors. Therefore, the extraction process is divided into two stages. First, the point cloud is filtered based on curvature to remove points with abnormally high curvature while preserving smooth and continuous depressed surfaces, as shown in Figure 7a. Second, a normal vector threshold is applied to further remove discrete points with abnormal orientations, as shown in Figure 7b. This two-stage filtering ensures the accuracy of the scour pit point cloud and provides reliable input for subsequent 3D reconstruction and volume calculation. It should be noted that curvature computation depends on the point cloud normals, so normal vectors must be calculated prior to curvature estimation.

For ∀ *i* ∈ [1, …, *n*], search for the point clouds *p_j_* (where *j* ∈ [1, …, *n*] in the pile-foundation scour-pit point cloud set ***P***, where the distance to point cloud *p_i_* ∈ ***P*** is less than *r*, and form the neighborhood point set ***N***(*p_i_*) of *p_i_*, i.e.,:

(20) $$N(p_{i})=\left\{\left\|p_{j}-p_{i}\right\|\leq r\right\}$$

Then, the covariance matrix *Cov_i_* of the point cloud *p_i_* is calculated as follows:

(21) $$Cov_{i}=\frac{1}{\left|N(p_{i})\right|}\sum (p_{j}-{\bar{p}}_{i}){\left(p_{j}-{\bar{p}}_{i}\right)}^{T}$$

where |·| denotes the cardinality of the set, i.e., the number of all points in the set; ${\bar{p}}_{i}$ is the centroid of the neighborhood point set of *p_i_*, which is calculated as follows:

(22) $${\bar{p}}_{i}=\frac{1}{\left|N(p_{i})\right|}\sum p_{j}$$

Finally, the covariance matrix *Cov_i_* is subjected to eigenvalue decomposition, yielding three eigenvalues *λ*_1_, *λ*_2_, and *λ*_3_. The eigenvector corresponding to the smallest eigenvalue, *n_i_* = (*n_x_*, *n_y_*, *n_z_*) *^T^*, is taken as the normal vector of the point cloud *p_i_*.

The curvature *C_i_* of the point cloud *p_i_* can be calculated based on the three eigenvalues as follows:

(23) $$C_{i}=\frac{\min(\lambda_{1},\lambda_{2},\lambda_{3})}{\lambda_{1}+\lambda_{2}+\lambda_{3}}$$

Following the above procedure, once the curvature of all points is obtained, point cloud filtering for the concave (scour pit) regions can be performed. Curvature-based filtering is employed to distinguish depressed regions from non-depressed regions based on the local geometric features of the point cloud. Depressed regions are typically smooth and exhibit low curvature, whereas noise or protruding regions have higher curvature. By removing points whose curvature exceeds a defined threshold, sharp or abnormal non-target points can be eliminated, thereby preserving continuous and smooth depressed surfaces and improving the accuracy of scour pit point cloud extraction. Specifically, points with curvature greater than a predefined threshold are removed, forming the filtered point cloud set ***P****_f_*. For effective and accurate extraction of scour-pit point clouds from the pile-foundation data, only points in ***P****_f_* whose normal vector components (*n_x_*, *n_y_*, *n_z_*) in the *x*-, *y*-, and *z*-directions exceed the corresponding directional thresholds *Tx*, *Ty* and *Tz* are retained. In this way, the initial valid scour-pit point cloud set ***P****_i_* is obtained.

### 4.2. Clustering and Morphology Restoration of Scour-Pit Point Clouds

Since the initial valid scour-pit point cloud set ***P****_i_* still contains environmental and terrain noise, for subsequent volume calculation, the study retains the bottom-layer points and performs vertical stacking to restore the morphology necessary for volume computation, thereby forming the target scour-pit point cloud. The bottom-layer points are extracted via depth-based descent detection along the *z*-coordinate direction of the point cloud. Specifically, points in ***P****_i_* exceeding a depth threshold are removed, keeping only points within the specified threshold, forming the bottom-layer point cloud set ***P****_b_*. Suppose there are s points in ***P****_b_*, i.e., ∀ *i* ∈ [1, …, *s*], *p_i_* ∈ ***P****_b_*, then:

(24) $$p_{i}=\left\{z_{i}\leq z_{\max}-\Delta z\right\}$$

where *z_i_* is the *z*-coordinate depth value of point *p_i_*; *z*_max_ is the depth of the highest point in ***P****_i_*; Δ*z* is the depth threshold.

**Figure 8:**
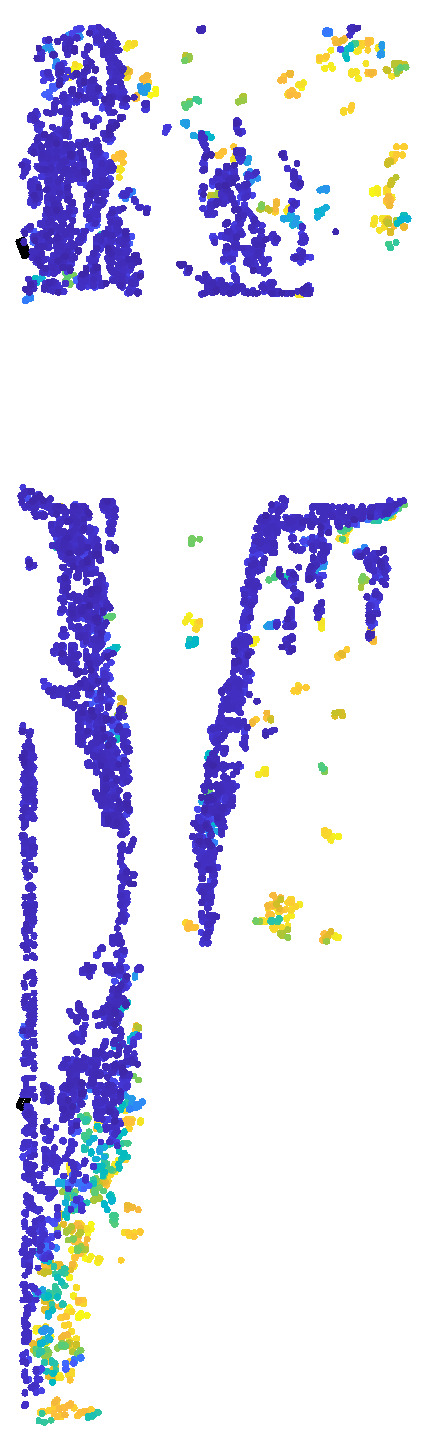
Bottom-layer scour-pit point clouds.

Figure 8 presents the extraction results of the bottom-layer scour-pit point cloud. After removing noise points and non-bottom-layer points, only those points closest to the seabed are retained, providing a more accurate representation of the true morphology and depth of the scour pits, and offering a reliable basis for subsequent 3D reconstruction and volume calculation.

To address interference among multiple scour pits during volume calculation, a density-based clustering method is applied to the bottom-layer scour-pit point cloud, resulting in multiple individual bottom-layer point clouds corresponding to each scour pit.

First, for ∀ *i*, *j* ∈ [1, …, *s*], *i* ≠ *j*, with *p_i_* ∈ ***P****_b_* and *p_j_* ∈ ***P****_b_*, the neighborhood set ***N***′(*p_i_*) of *p_i_* is determined as follows:

(25) $$N^{\prime }(p_{i})=\left\{p_{j}|\left\|p_{j}-p_{i}\right\|\leq \varepsilon \right\}$$

where *ε* is the threshold distance for neighboring points.

Next, the neighborhood sets of all points in the bottom-layer point cloud set ***P****_b_* are determined. Points whose neighborhood contains more than a preset minimum number *M* of points are defined as core points. Suppose there are *γ* core points forming the set ***P****_γ_*; these core points satisfy:

(26) $$\forall o\in \left[1,\ldots ,\gamma \right],\mathrm{sum}(N^{\prime }(p_{o}))\geq M$$

where sum(·) denotes the count of points in the point cloud.

Finally, clustering of the core points is performed to obtain individual bottom-layer point clouds. For ∀ *o* ∈ [1, …, *γ*], the core point *p_o_* is first taken as the initial point of cluster ***A***, i.e., ***A*** = {*p_o_*}. Then, the neighborhood ***N***_∈_(*p_o_*) of $p_{o}$ is constructed within the set ***P****_γ_*, and any core point in this neighborhood not yet in cluster ***A*** is added to ***A***, i.e., ***A***←***A*** ∪ *p_o_*. The same procedure is then repeated for each newly added core point in ***A***, constructing its neighborhood and adding any non-***A*** core points, until no more core points can be added, completing the final clustering of ***A***. After cluster ***A*** is formed, remaining non-***A*** core points in ***P****_γ_* are selected, and the above procedure is repeated to form clusters ***B***, ***C***, …, thereby generating the bottom-layer point cloud dataset ***P****_c_* consisting of several clusters.

To accurately compute the volume of the scour pits, complete point clouds must be obtained through morphological restoration. For each cluster, points are stacked along the *z*-axis to reconstruct the full scour-pit point cloud. Specifically, the number of stacking layers *n* for each cluster is determined based on the *z*-coordinate depth values of the points within that cluster:

(27) $$n=\frac{z_{\max}-\bar{z}}{\Delta z}$$

where Δ*z* is the depth increment between layers, and $\bar{z}$ is the average depth of the entire point cloud.

Then, based on the number of expansion layers, the points of each cluster are stacked vertically along the *z*-axis. Assume the point clouds in cluster ***A*** are *p_l_* = (*x_l_*, *y_l_*, *z_l_*), ∀ *l* ∈ [1, *ζ*]. For all points in cluster ***A***, *n* new layers of point clouds are created. The *z*-coordinate of the points in each layer is increased by *k*·Δ*z*, resulting in the *k*-th layer point cloud set ***A****^k^*:

(28) $$A^{k}=\left\{x_{l},y_{l},z_{l}+k\Delta z\right\}$$

where *k* is the current layer number, with *k* = 1, 2, …, *n*.

The point clouds from each layer are then merged to form the final target point cloud dataset ***A****^f^*:

(29) $$A^{f}=A\cup A^{1}\cup A^{2}\cup \ldots \cup A^{k}$$

After the stacking extension of cluster ***A*** is completed, the same procedure is applied to all clusters, ultimately yielding the morphology-restored initial scour-pit point cloud dataset ***P****_r_*.

Since the morphology-restored model may contain scaling errors, the points in the scour-pit point cloud set ***P****_r_* are scaled to correct these discrepancies, resulting in the final corrected scour-pit point cloud dataset ***P****_f_*. The scale correction parameter *s* is defined as the ratio of the actual seabed depth to the maximum depth of the 3D reconstructed point cloud, and is used to convert the relative depths in the point cloud into actual depths:

(30) $$s=\frac{z_{b}}{z_{\max}}$$

where *z_b_* is the actual seabed depth.

**Figure 9:**
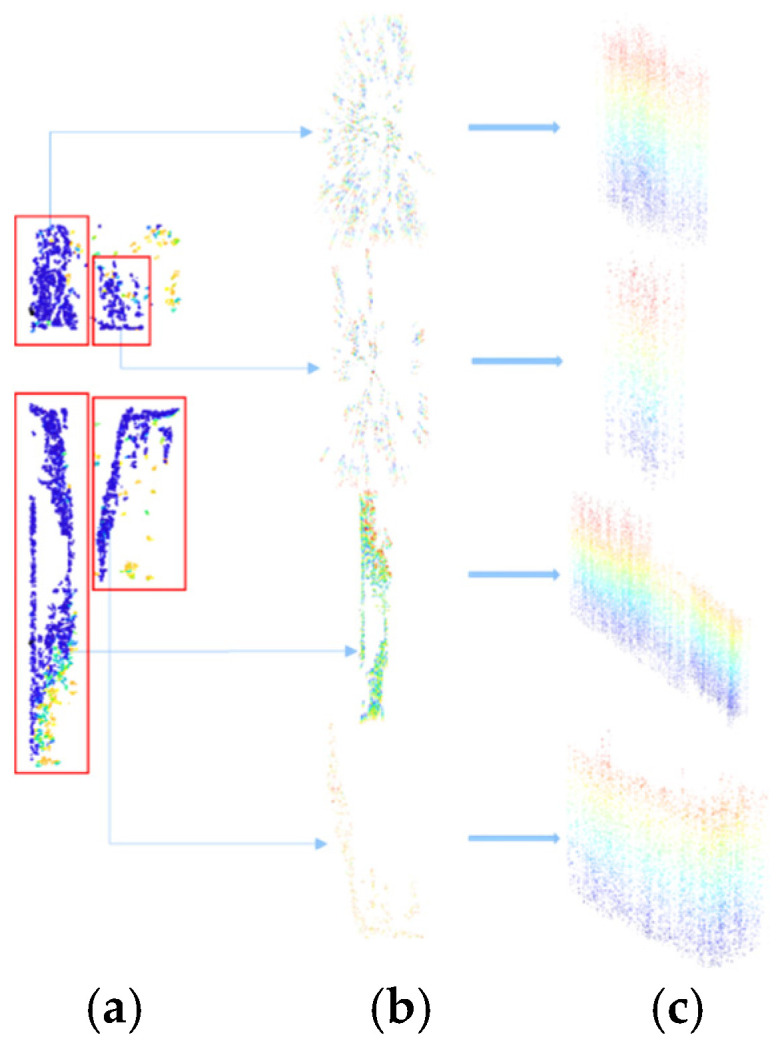
Corrected scour pit point clouds: (**a**) bottom layer scour pit point clouds; (**b**) corrected top-down point clouds view; (**c**) corrected side view point clouds.

Figure 9 shows the final corrected scour pit point cloud.

## 5. Volume Calculation of Scour Pits Around Offshore Wind Turbine Pile Foundations

To accurately assess the scour condition of the pile foundation, the volume of the scour-pit point cloud is calculated for quantitative analysis. The AlphaShape algorithm [21] constructs tetrahedra from the boundary points of the point cloud based on a shape control parameter *α*, which is particularly advantageous for generating closed surface models and enables precise volume measurement. Specifically, the algorithm generates multiple tetrahedra by connecting four adjacent boundary points of the scour pit and computes the total volume by summing the volumes of all tetrahedra. The smoothness and level of detail of the tetrahedra can be adjusted via the parameter *α*, further enhancing the accuracy of the final volume calculation.

**Figure 10:**
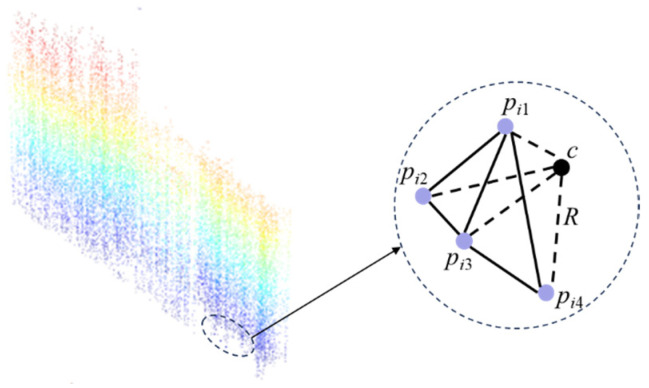
Circumcenter *c* of the four vertices of the tetrahedron.

The model constructed from the corrected scour-pit point cloud dataset ***P****_f_* represents a shell structure, meaning it contains no interior points. Therefore, it can be directly treated as the boundary point cloud of the scour pit. Let the set of tetrahedra generated from the point cloud ***P****_f_* be *T* = (*T*_1_, *T*_2_, …, *T_i_*), where each tetrahedron *T_i_* is composed of four points, i.e., *T_i_* = (*p_i_*_1_, *p_i_*_2_, *p_i_*_3_, *p_i_*_4_). These four points clouds are the four vertices of the tetrahedron, with coordinates *p_i_*_1_ = (*x_i_*_1_, *y_i_*_1_, *z_i_*_1_), *p_i_*_2_ = (*x_i_*_2_, *y_i_*_2_, *z_i_*_2_), *p_i_*_3_ = (*x_i_*_3_, *y_i_*_3_, *z_i_*_3_), and *p_i_*_4_ = (*x_i_*_4_, *y_i_*_4_, *z_i_*_4_). The circumcenter *c* of these four vertices is calculated as shown in Figure 10:

(31) $$c=A^{-1}B={\left[\begin{array}{ccc} x_{i2}-x_{i1} & y_{i2}-y_{i1} & z_{i2}-z_{i1}\\ x_{i3}-x_{i1} & y_{i3}-y_{i1} & z_{i3}-z_{i1}\\ x_{i4}-x_{i1} & y_{i4}-y_{i1} & z_{i4}-z_{i1} \end{array}\right]}^{-1}\left[\begin{array}{c} {x_{i2}}^{2}+{y_{i2}}^{2}+{z_{i2}}^{2}-{x_{i1}}^{2}-{y_{i1}}^{2}-{z_{i1}}^{2}\\ {x_{i3}}^{2}+{y_{i3}}^{2}+{z_{i3}}^{2}-{x_{i1}}^{2}-{y_{i1}}^{2}-{z_{i1}}^{2}\\ {x_{i4}}^{2}+{y_{i4}}^{2}+{z_{i4}}^{2}-{x_{i1}}^{2}-{y_{i1}}^{2}-{z_{i1}}^{2} \end{array}\right]$$

The circumradius *R* of each tetrahedron is determined based on the calculated circumcenter *c* = (*x_ic_*, *y_ic_*, *z_ic_*):

(32) $$R=\frac{\sqrt{{\left(x_{i1}-x_{ic}\right)}^{2}+{\left(y_{i1}-y_{ic}\right)}^{2}+{\left(z_{i1}-z_{ic}\right)}^{2}}}{2}$$

Since the circumradius *R* of a tetrahedron is closely related to the spatial distribution of its four vertices, a smaller R indicates that the points are closer together, implying a denser local point cloud. The boundary geometry of the scour pit is formed by dense point clouds, so the corresponding tetrahedra generally have smaller circumradii. In contrast, tetrahedra with larger circumradii are typically composed of sparse or noisy points, which may fail to accurately represent the scour pit boundary. Therefore, in this study, tetrahedra are selected based on *R*, retaining only those with *R* ≤ *α*; the final set of retained tetrahedra constitutes the three-dimensional model of the scour pit.

Due to noise interference and uneven sampling in the point cloud data, some tetrahedra may exhibit elongated or distorted shapes. To mitigate this issue, a weight factor *w_i_* is introduced to adjust the volume contribution of each tetrahedron. The total scour-pit volume is then obtained by summing the volumes of all tetrahedra:

(33) $$V=\sum_{i=1}^{n}\frac{1}{6}w_{i}\left|\begin{array}{ccc} x_{i2}-x_{i1} & y_{i2}-y_{i1} & z_{i2}-z_{i1}\\ x_{i3}-x_{i1} & y_{i3}-y_{i1} & z_{i3}-z_{i1}\\ x_{i4}-x_{i1} & y_{i4}-y_{i1} & z_{i4}-z_{i1} \end{array}\right|$$

where *w_i_* is the ratio of the shortest edge *L^i^*_min_ to the longest edge *L^i^*_max_ of tetrahedron *i*:

(34) $$w_{i}=\frac{L_{\min}^{i}}{L_{\max}^{i}}$$

## 6. Numerical Testing of Simulated Scour Environments

To verify the accuracy of the proposed 3D reconstruction and volume detection method for wind turbine foundation scour based on side-scan sonar, numerical tests were conducted in five simulated environments, encompassing three scenarios: single scour pit, double scour pits, and triple scour pits. The procedure begins with creating simulated models in SolidWorks (2024 version), which are then converted into point clouds using CloudCompare (v2.14. alpha version). Subsequently, simulated 2D seabed side-scan sonar images are generated by adding light sources and adjusting the illumination direction on the point cloud data, providing inputs for further analysis.

### 6.1. Test of Single Scour Pit Simulation Environment

**Figure 11:**
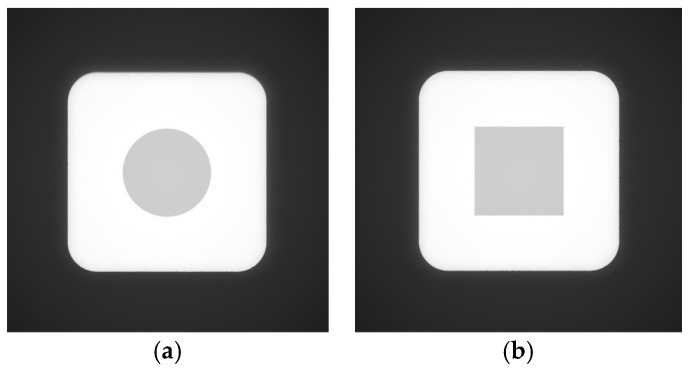
Grayscale images of side-scan sonar simulation environments containing single scour pits: (**a**) grayscale image containing a cylindrical scour pit; (**b**) grayscale image containing a square scour pit.

For the single scour pit simulation, two pit geometries were selected: cylindrical and square. After model creation, point cloud generation, and the addition of light sources and illumination direction, the resulting grayscale images of the simulated side-scan sonar environments containing the single scour pits are shown in Figure 11.

**Figure 12:**
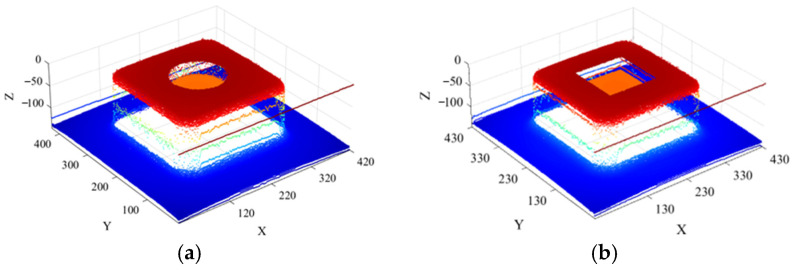
Three-dimensional reconstruction of side-scan sonar simulation environments con-taining single scour pits: (**a**) 3D reconstruction of the environment containing a cylindrical scour pit; (**b**) reconstruction of the environment containing a square scour pit.

Since the two grayscale images of the simulated side-scan sonar environments containing single scour pits do not include invalid shadow regions, they can be directly reconstructed in 3D using the SFS algorithm. The resulting 3D reconstruction images of the simulated single scour pit environments are presented sequentially in Figure 12.

**Figure 13:**
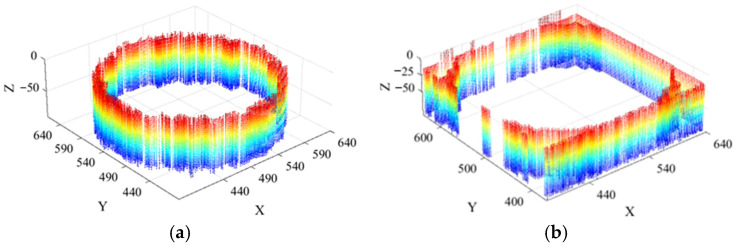
Point cloud of single scour pits: (**a**) cylindrical scour pit; (**b**) square scour pit.

Since each of the aforementioned pile foundations contains only a single scour pit in the 3D-reconstructed point cloud data, the scour pit obtained after dual extraction using curvature and normal vectors, along with retention of the bottom-layer points, does not require clustering. It can be directly subjected to point cloud shape restoration, resulting in a complete scour-pit point cloud, as shown in Figure 13.

The theoretical and detected volumes of the scour pits are compared in Table 1. As shown, the proposed algorithm achieves very high detection accuracy when processing single-scour-pit point cloud data, with an average error of only 0.34%.

### 6.2. Test of Double Scour-Pit Simulation Environment

**Figure 14:**
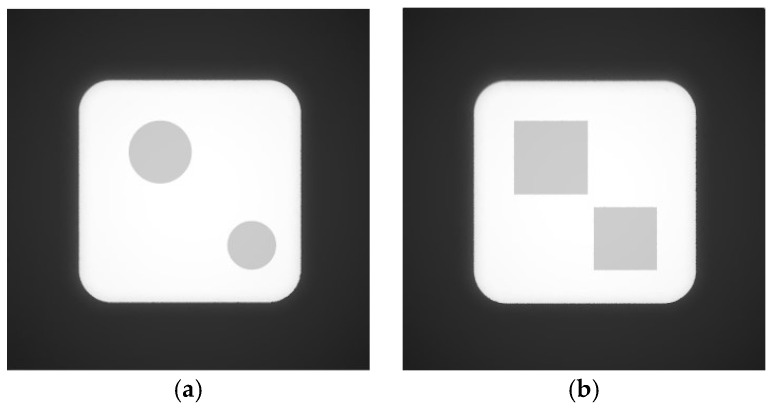
Grayscale images of the side-scan sonar simulation environment with two scour pits: (**a**) grayscale image with two cylindrical scour pits; (**b**) grayscale image with two rectangular scour pits.

To further evaluate the accuracy of the scour detection algorithm, a numerical test was conducted in an environment containing two scour pits. Figure 14 presents the grayscale images of two cylindrical scour pits of different sizes and two rectangular scour pits of different sizes, respectively.

**Figure 15:**
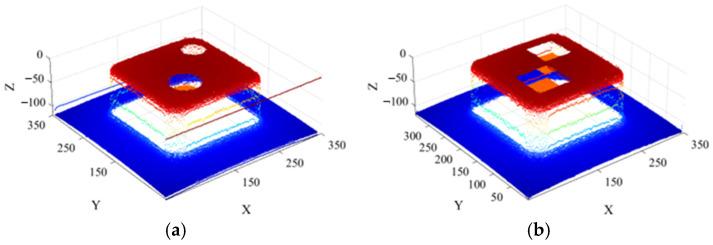
Three-dimensional reconstruction of the side-scan sonar simulation environment with two scour pits: (**a**) 3D reconstruction of cylindrical scour pits; (**b**) 3D reconstruction of square scour pits.

Similarly, 3D reconstruction was directly performed using the SFS algorithm, resulting in the 3D reconstruction of the simulated side-scan sonar environment containing two scour pits, as shown in Figure 15.

**Figure 16:**
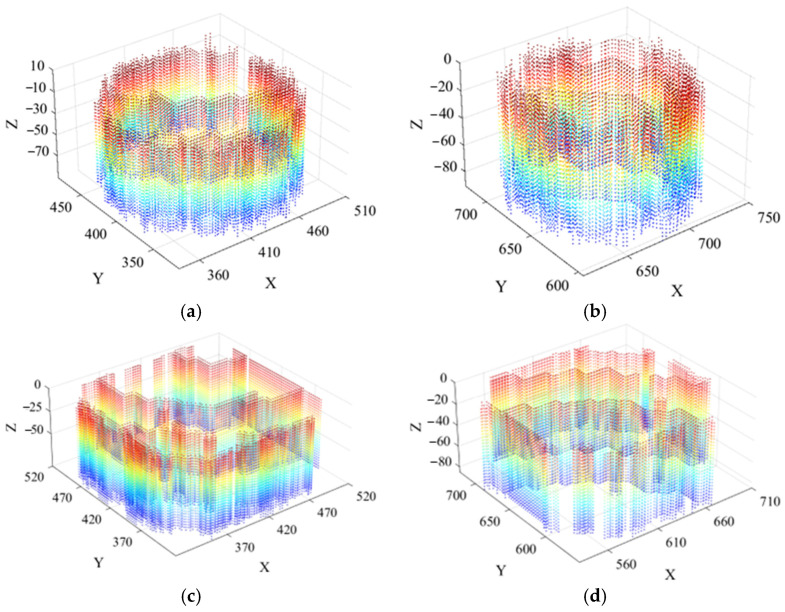
Point clouds of double scour pits: (**a**) large cylindrical scour pit; (**b**) small cylindrical scour pit; (**c**) large square scour pit; (**d**) small square scour pit.

To enable independent and accurate volume detection of each scour pit in the model, the scour pit point clouds were first extracted using both curvature and normal vector information. Subsequently, depth-based descent detection and clustering along the *z*-coordinate were performed to obtain the bottom-layer scour pit point clouds. Finally, point cloud layer stacking was applied for morphological restoration, resulting in the final scour pit point cloud dataset, as shown in Figure 16.

Based on the final scour pit point cloud dataset, the volumes of the scour pits were calculated using the AlphaShape algorithm, as shown in Table 2. As indicated in the table, the maximum detection error among the four independent scour pits in the two environments is 5.80%, with an average detection error of 4.775%, demonstrating that the proposed algorithm can effectively and accurately compute volumes in double-scour-pit model environments. These results highlight the robustness and reliability of the method, indicating its potential applicability for more complex multi-pit scour scenarios commonly encountered in real offshore wind turbine foundation sites.

### 6.3. Test of Triple Scour Pit Simulation Environment

**Figure 17:**
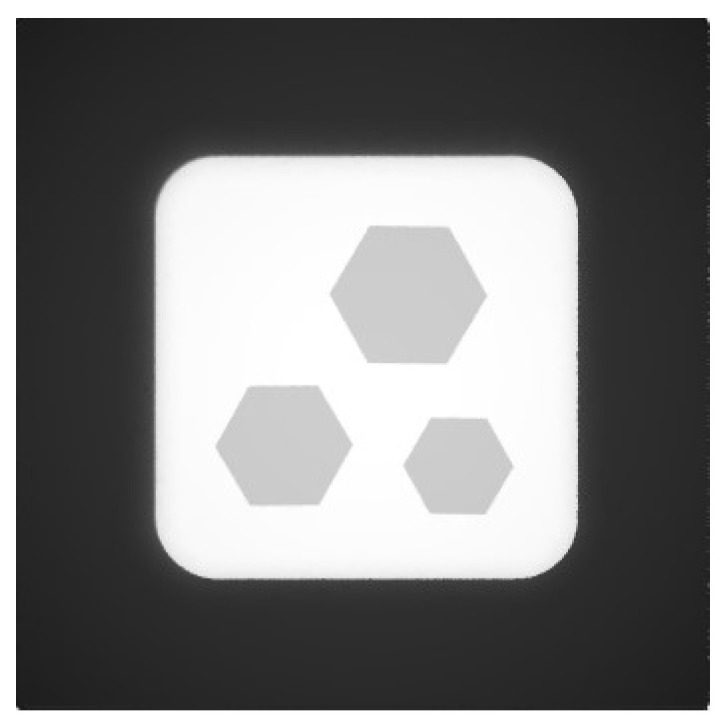
Grayscale images of the side-scan sonar simulation environment containing three scour pits.

Figure 17 presents the 2D grayscale images of three hexagonal-prism scour pits of different sizes, obtained after modeling, point cloud generation, addition of light sources, and adjustment of illumination directions.

**Figure 18:**
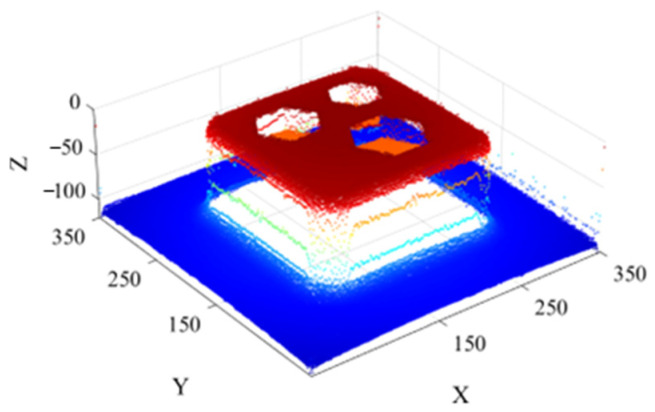
Three-dimensional reconstruction of the side-scan sonar simulated environment with three scour pits.

Figure 18 shows the three-dimensional reconstruction of the three scour pits generated using the SFS algorithm, clearly illustrating the spatial distribution and geometric features of each scour pit based on the 2D side-scan sonar images.

**Figure 19:**
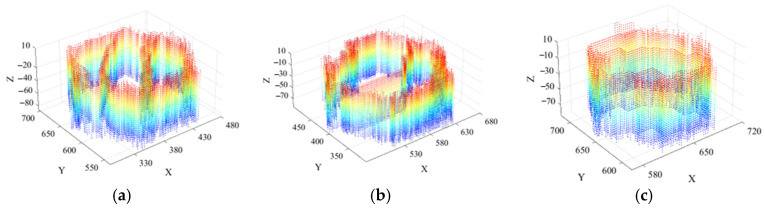
Scour pit point clouds for three scour pits: (**a**) large hexagonal prism scour pit; (**b**) medium hexagonal prism scour pit; (**c**) small hexagonal prism scour pit.

Figure 19 presents the scour pit point cloud data obtained after dual extraction based on curvature and normal vectors, depth-based descent detection and clustering along the *z*-coordinate, and point cloud morphological restoration.

Table 3 presents the volume detection comparison for the three scour pits. As shown, the volumes detected by the algorithm are very close to the theoretical values, with a maximum error of 4.3% and an average error of 2.4%, demonstrating that the proposed scour pit volume detection algorithm is both effective and highly accurate in multi-scour-pit environments.

Through multi-scenario experiments, the proposed method achieved an average detected scour pit volume of 97.495% relative to the theoretical values, demonstrating robust performance across different scour morphologies, with measurement errors consistently controlled within 6% of the true values.

To validate the effectiveness and computational efficiency of the proposed method for scour pit volume estimation, comparative experiments were conducted focusing on the volume calculation of scour pits. Side-scan sonar images of a single square scour pit were selected as the test data for the comparison, and the experimental results are presented in Table 4.

Method 1 is a volume calculation approach based on regular grid integration [22]. First, the 3D point cloud corresponding to the single scour pit is projected onto the XY plane, and a regular 2D grid is constructed on this plane. The *z*-values of the point cloud are then interpolated to generate an elevation distribution on the regular grid, with the maximum elevation taken as the reference plane. By integrating the height differences within each grid cell, the estimated volume of the scour pit is obtained.

Method 2 is a volume estimation approach based on point cloud slicing [23]. The scour pit point cloud is sliced into layers of equal thickness along the z-direction. For each slice, the points are projected onto the XY plane, and the corresponding cross-sectional area is calculated using the convex hull algorithm. By integrating the cross-sectional areas of all slices along the *z*-direction, the overall volume of the scour pit is obtained.

As shown in Table 4, Method 1, based on regular grid integration, tends to underestimate the volume due to the smoothing effect of interpolation and requires a relatively long computation time. Method 2, based on point cloud slicing, may produce deviations in volume estimation when depressions are present because the convex hull calculation can introduce an envelope effect, although its computation time is shorter. In contrast, the proposed method achieves a favorable balance between accuracy and efficiency: it maintains a high level of volume estimation precision while keeping computation time relatively short. This allows for a more realistic representation of the true 3D geometry of scour pits and provides a rapid and reliable approach for assessing seabed scour pit volumes in practical engineering applications.

## 7. Experimental Testing in Real Scour Environments

**Figure 20:**
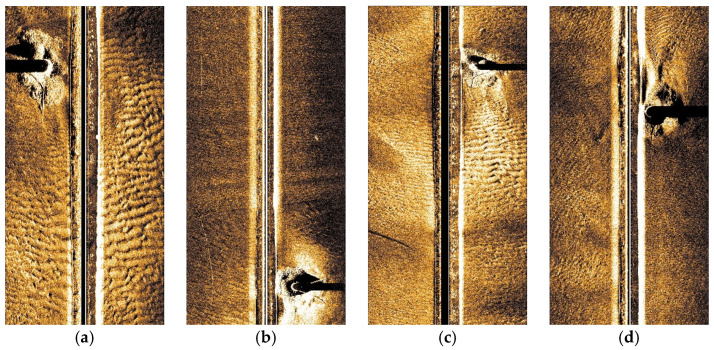
Four sets of actual side-scan sonar images: (**a**) 1# pile; (**b**) 2# pile; (**c**) 3# pile; (**d**) 4# pile.

Although the above numerical test results have verified the effectiveness of the proposed detection algorithm, its performance in real-world scour environments still requires further validation. Therefore, additional experimental tests were conducted on side-scan sonar images of pile foundations at the Zhanjiang Xuwen Offshore Wind Farm, as shown in Figure 20.

**Figure 21:**
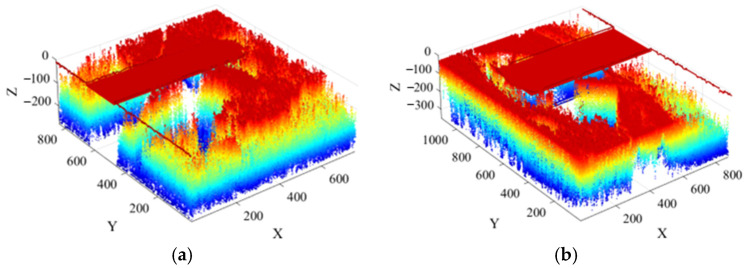

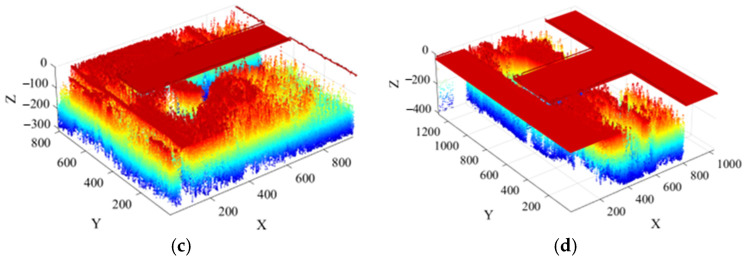
Actual 3D pile foundation point clouds: (**a**) 1# pile; (**b**) 2# pile; (**c**) 3# pile; (**d**) 4# pile.

To facilitate the effective extraction of scour regions around the pile foundations, the sonar images of the piles were first reconstructed in 3D using the SFS method, resulting in the three-dimensional point cloud data shown in Figure 21. The figure clearly illustrates the scour depth information, with blue points indicating the extent of scour around the offshore wind turbine pile foundations.

**Figure 22:**
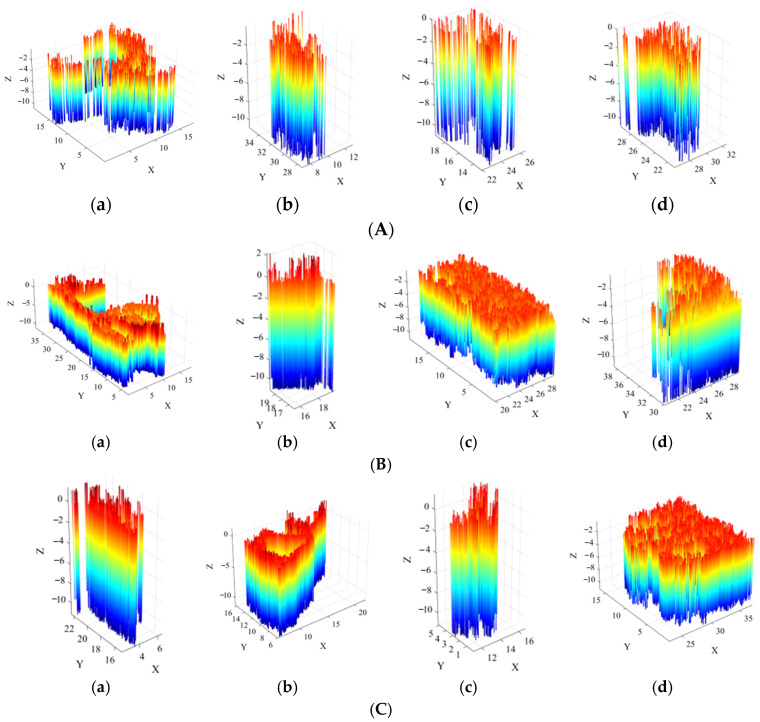

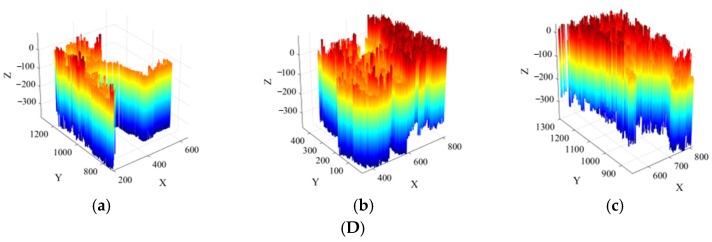
Four sets of individual scour pit point clouds: (**A**) 1# pile foundation scour pits; (**B**) 2# pile foundation scour pits; (**C**) 3# pile foundation scour pits; (**D**) 4# pile foundation scour pits; (**a**) scour pit 1; (**b**) scour pit 2; (**c**) scour pit 3; (**d**) scour pit 4.

The scour pit point cloud data was then obtained through dual extraction based on curvature and normal vectors. Depth descent detection and clustering were performed along the *z*-coordinate to form individual bottom-layer scour pit point clouds. For each independent scour pit point cloud, point cloud morphology was restored using the layered extension method, resulting in the scour pit point cloud data shown in Figure 22. The figure sequentially presents the individual scour pit point clouds for each pile foundation.

Finally, Table 5 presents the volume calculation results for the four scour pits of each pile foundation. As shown, significant differences in scour pit volumes exist among different pile foundations; for example, the first scour pit of Pile #2 has the largest volume (5.89 m^3^), while the second and fourth pits are smaller (0.12 m^3^ and 0.66 m^3^, respectively). Within the same pile, substantial variations in volumes among different scour pits are also observed, reflecting the non-uniformity of the scouring process and the influence of pile location on scour formation. Overall, the algorithm can accurately extract the spatial characteristics of each scour pit and quantify their volumes, providing reliable data support for scour risk assessment and protective design.

The test results in the actual scour pit environment indicate that the proposed side-scan sonar-based method for three-dimensional reconstruction and volume detection of offshore wind turbine pile foundations can accurately reconstruct the seabed piles and reliably measure the scour pit volumes of the wind turbine foundations.

## 8. Conclusions

Accurate detection of scour pit volumes is of great significance for the maintenance and management of offshore wind turbine pile foundations. Based on the acquired side-scan sonar images of pile foundations, this study sequentially obtains effective grayscale images of pile scour pits, performs three-dimensional reconstruction, extracts multiple individual scour pit point clouds, and calculates scour pit volumes, thereby proposing a method for 3D reconstruction and volume detection of pile foundation scour pits. Based on numerical simulations and experimental tests, the following conclusions can be drawn:

(1) By performing connected component analysis and regional feature extraction on pile foundation side-scan sonar images, the proposed method can effectively identify and extract the pile foundation regions, thereby improving the efficiency of subsequent scour detection;

(2) Using the SFS algorithm for iterative optimization of the pile region images, three-dimensional reconstruction is achieved to obtain 3D pile point cloud data. Through curvature and normal vector filtering, *z*-coordinate depth descent detection, clustering, and morphological restoration, precise acquisition of scour pit point clouds is realized;

(3) By applying the AlphaShape algorithm with a weighting factor to each individual scour pit point cloud, more accurate scour pit volumes are obtained.

Compared with current manual diving and sonar inspection methods, the side-scan sonar–based three-dimensional reconstruction and volume detection of offshore wind turbine pile foundation scours not only enables the acquisition of effective scour pit regions but also allows for precise calculation of scour volumes. This approach provides an advanced technical solution for the management and maintenance of offshore wind turbine foundations, with broad practical application value.

Although the proposed method achieves good detection accuracy in both numerical simulations and field data from offshore wind farms, it may still face challenges under complex marine environmental conditions. For instance, turbid water and suspended particles can enhance sonar signal attenuation and scattering, reduce the contrast of side-scan sonar images and affect the grayscale distinction between scour areas and the surrounding seabed. In seabed environments with significant morphological variations, acoustic shadows may locally resemble actual scour regions, increasing the difficulty of region discrimination. To address these issues, future research could consider incorporating multi-source data fusion strategies (e.g., combining multibeam bathymetry data or inertial navigation information) and developing an adaptive parameter adjustment mechanism based on local grayscale and texture features. Additionally, exploring feature discrimination methods assisted by learning-based strategies may further enhance the algorithm’s robustness under varying sea conditions and imaging scenarios, improving its ability to distinguish scour regions from acoustic shadows in complex environments.

## Figures and Tables

**Table 1:** Detection results of single scour pits.

Name	Theoretical Scour-Pit Volume (m^3^)	Detected Scour-Pit Volume (m^3^)	Detection Error (m^3^)	Error Percentage (%)
Cylindrical	4.948	4.944	0.004	0.08
Square	6.3	6.262	0.038	0.6

**Table 2:** Double scour pit detection results.

Name	Theoretical Scour-Pit Volume (m^3^)	Detected Scour-Pit Volume (m^3^)	Detection Error (m^3^)	Error Percentage (%)
Large Cylindrical	2.199	2.327	0.128	5.8%
Small Cylindrical	1.407	1.324	0.083	5.8%
Large Square	3.703	3.586	0.117	3.2%
Small Square	2.800	2.678	0.122	4.3%

**Table 3:** Detection results for three scour pits.

Name	Theoretical Scour-Pit Volume (m^3^)	Detected Scour-Pit Volume (m^3^)	Detection Error (m^3^)	Error Percentage (%)
Large Hexagonal	3.073	3.096	0.023	0.7%
Medium Hexagonal	2.405	2.352	0.053	2.2%
Small Hexagonal	1.641	1.57	0.071	4.3%

**Table 4:** Comparative experimental results of the two volume calculation methods.

Algorithm	Detected Scour-Pit Volume (m^3^)	Detection Error (%)	Computational Efficiency (s)
Proposed Method	6.262	0.6	0.043
Method 1	3.07	52.2	7.009
Method 2	5.05	19.8	0.026

**Table 5:** Detection results of four pile foundation scour pits.

Name	Scour Pit 1 (m^3^)	Scour Pit 2 (m^3^)	Scour Pit 3 (m^3^)	Scour Pit 4 (m^3^)
1# Pile	2.4	0.32	0.26	0.46
2# Pile	5.89	0.12	1.95	0.66
3# Pile	0.26	1.21	0.19	2.19
4# Pile	3.73	4.16	4.17	None

## Data Availability

The data is included in the article.
